# Coblator-Assisted Endoscopic Transnasal Resection of a Large Nasopharyngeal Pleomorphic Adenoma

**DOI:** 10.1155/2019/4654357

**Published:** 2019-02-27

**Authors:** Jonathan Bowman, Anu Daudia, Neil Sahasrabudhe, Antonio Belloso

**Affiliations:** ^1^University of Manchester, Manchester, UK; ^2^Royal Blackburn Teaching Hospital, Blackburn, UK

## Abstract

**Background:**

Pleomorphic adenomas occurring in the adult nasopharynx are rare, with our literature search identifying only 11 previous English-language reports. We document the unusual case of a large nasopharyngeal pleomorphic adenoma that was resected using radiofrequency coblation via an endoscopic transnasal approach.

**Methods:**

A 39-year-old male presented with worsening nasal congestion, intermittent otalgia, and a progressive change in voice. Flexible nasendoscopy showed a large homogeneous mass occupying the postnasal space, and computed tomography confirmed a 28 × 31 × 22 mm nasopharyngeal tumour. The biopsy-proven benign tumour was locally dissected using a coblator-assisted transnasal approach.

**Results:**

Histology confirmed complete excision of a myoepithelial-rich pleomorphic adenoma. The patient was symptom-free postoperatively, and no signs of recurrence were seen at one-year follow-up.

**Conclusions:**

This is a useful addition to the existing literature on surgical procedures used to treat benign pathology in the nasopharynx. The minimally invasive technique was well tolerated and had favourable patient outcomes.

## 1. Introduction

Pleomorphic adenomas are benign neoplasms, with diverse morphologies, comprised of variable proportions of epithelial and myoepithelial elements embedded within a mucopolysaccharide stroma [1]. They are the most common tumour of the major salivary glands, with most cases presenting within the superficial lobe of the parotid gland; however, they can occur at any site where the salivary tissue is found. Despite the presence of many minor glands in the upper aerodigestive tract, pleomorphic adenomas occurring in the adult nasopharynx are rare. Our literature search identified only 11 previous English-language reports, summarised in Table 1 [2–12].

Although benign, pleomorphic adenoma can be locally invasive and untreated tumours may grow to exaggerated proportions. Pleomorphic adenoma can also undergo malignant change to become the malignancy, carcinoma ex pleomorphic adenoma [13]. For these reasons, and because lesion longevity is considered a risk factor for malignant transformation, the complete surgical excision of pleomorphic adenoma is recommended [14, 15]. When accurately diagnosed, and appropriately treated, pleomorphic adenoma should not recur, and prognosis is good.

We document the endoscopic, radiographic, and histopathological features of a large nasopharyngeal pleomorphic adenoma resected using a coblator-assisted transnasal approach. We discuss our case within the context of the other cases reported in the literature; however, to the best of our knowledge, this is the first published description of this pathology to be treated with this surgical technique.

## 2. Case Report

A 39-year-old male, with no significant medical history, presented to our Otolaryngology Department. He reported several months of worsening nasal congestion, intermittent otalgia, and a progressive change in voice.

Clinical examination confirmed bilateral obstruction of the nasal airway and showed the patient was a habitual mouth breather. Diagnostic flexible nasendoscopy demonstrated significant hypertrophy of the turbinates and the presence of a large homogeneous non-indurated soft tissue mass occupying the nasopharynx.

**Figure 1:**
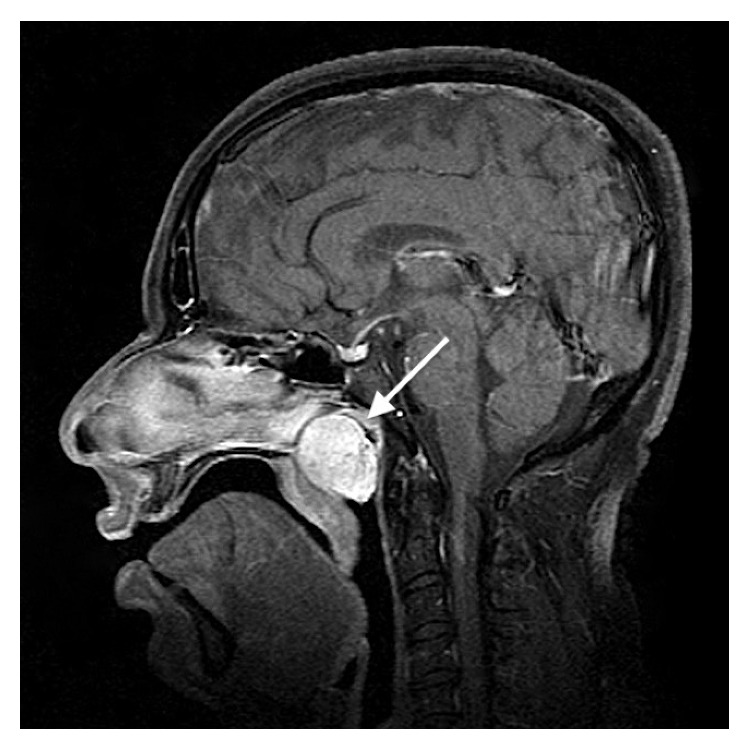
Sagittal T1-weighted MR image showing a well-defined enhancing tumour (arrow) completely filling the nasopharynx.

Computed tomography (CT) showed a well-defined 28 × 31 × 22 mm tumour in the nasopharynx obstructing both Eustachian tube orifices, but with no signs of local or regional lymphadenopathy. Contrast magnetic resonance (MR) imaging (Figure 1) demonstrated a uniformly enhancing pedunculated polypoid mass that displaced the soft palate inferiorly, but without evidence of skull base involvement.

**Figure 2:**
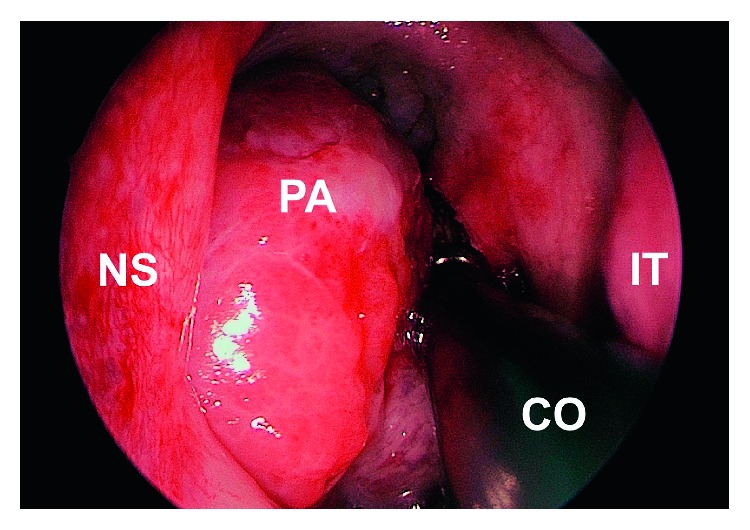
Intraoperative 0-degree endoscope view showing pleomorphic adenoma resection using the coblator device. NS, nasal septum; PA, pleomorphic adenoma; CO, coblator; IT, inferior turbinate.

**Figure 3:**
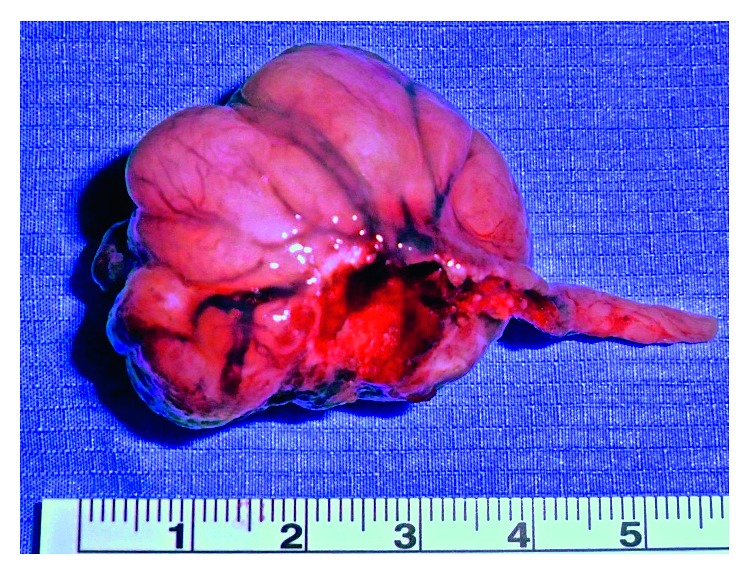
Clinical photograph of the unfixed surgical specimen (scale: cm).

Following an incisional biopsy that showed histological features of pleomorphic adenoma, the multidisciplinary team consensus was to offer surgery to resect the entire tumour for definitive histology. The patient was consented and operated using an endoscopic transnasal approach under general anaesthesia. Standard functional endoscopic surgery instrumentation was used, in a procedure assisted by a radiofrequency coblator device (Smith & Nephew Inc., London, United Kingdom). The tumour's pedicle was identified endoscopically as originating from the left lateral nasopharynx and, using coblation, was carefully dissected en bloc from its mucosal attachment (Figure 2) leaving the wound bed to heal by secondary intention. Finally, the specimen (Figure 3) was delivered transorally with forceps. The procedure was completed uneventfully as a day case without complication.

**Figure 4:**
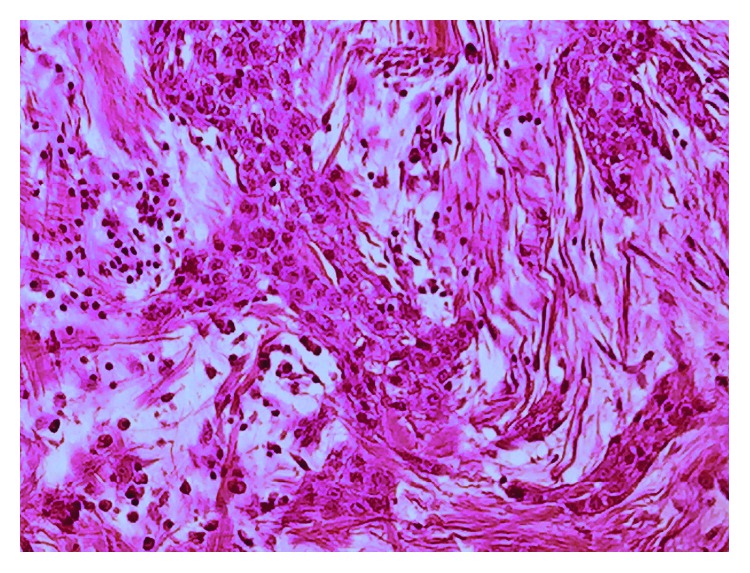
Photomicrograph showing pleomorphic adenoma histology (hematoxylin and eosin).

Definitive histology confirmed the complete excision of a myoepithelial-rich pleomorphic adenoma with negative resection margins. Microscopic sections (Figure 4) showed a circumscribed non-encapsulated tumour with no evidence of perineural or lymphovascular invasion. Immunohistochemical stains were strongly positive for cytokeratin AE1/3, CK5/6, and p63 (with a small portion reverse staining with CK7) and positive for vimentin; however, stains were negative for smooth muscle actin, S100 protein, SOX-10, and CD10. The Ki-67 proliferative index was low at <2%.

The patient was symptom-free postoperatively, and follow-up flexible nasendoscopy at one year demonstrated no signs of local recurrence.

## 3. Discussion

Pleomorphic adenomas occurring in the adult nasopharynx are rare. Our literature review identified only 11 previously published case reports; the mean age of patients affected was 56.8 years (range 29–80 years) with a male : female distribution of 3 : 8 [2–12]. The presenting symptoms most frequently reported were nasal obstruction, hearing loss, aural fullness, otalgia, and changes of voice. In common with the previously reported cases, our 39-year-old male patient described symptoms of nasal obstruction, otalgia, and voice change.

We evaluated our patient's tumour with diagnostic endoscopy, cross-sectional imaging, and tumour biopsy. Imaging findings were documented in nine previous cases [3, 5–12]. The imaging modalities used were either CT or MR, and three cases utilised both CT and MR in lesion evaluation [3, 10, 12]. No reports documented any evidence of tumour infiltration. Preoperative biopsy was undertaken in seven previous cases, five confirmed pleomorphic adenoma on histology, whereas two utilised a fine needle aspiration technique [3, 5, 6, 9–12]. Cytology correctly identified a pleomorphic adenoma in one case; however, it was suggestive of squamous cell carcinoma in the second (leading to that patient receiving inappropriate radical chemoradiotherapy before later biopsy showed a pleomorphic adenoma on histology). Our case demonstrated a preoperative clinico-radio-pathological correlation; clinical and imaging findings appeared benign, and subsequent tumour biopsy provided a tissue diagnosis of pleomorphic adenoma.

The large size and postnasal location of our patient's pleomorphic adenoma made the choice of surgical access an important consideration. Open approaches to the nasopharynx, extensively summarised by de Almeida et al. [5], can offer excellent surgical exposure; however, these accesses involve significant soft tissue disruption and may necessitate bone removal. A Le Fort I osteotomy may also facilitate cosmetic and bilateral access to the nasopharynx, although we were uncertain whether it would allow the manoeuvrability required for resection in this case. The majority of the previously documented cases used endoscopic transnasal approaches, variously combined with transoral accesses, for tumour resection [3–5, 7–11]. Similarly to those cases, we used an endoscopic transnasal approach to avoid the significant morbidities of facial deformity, disruption of the nasal skeleton, dental malocclusion, and also to reduce the risk of iatrogenic Eustachian tube damage. No complications were encountered in our patient's management; however, those surgical complications previously reported included soft palate paresis and nasal speech (open transmandibular transpterygoid approach) and Eustachian tube dysfunction (unspecified endoscopic-guided approach) [2, 4].

We chose to use a radiofrequency coblator for tumour resection. Coblation is widely used in adenoidectomy, tonsillectomy, and inferior turbinoplasty; however, applications have also been demonstrated in the resection of other benign pathologies, including juvenile angiofibroma and nasopharyngeal cysts [16, 17]. The advantage of using coblation is the ability to obtain excellent haemostasis, whilst inflicting minimal thermal damage, and this may minimise postoperative pain and promote early wound healing. We found a coblator-assisted dissection straightforward to perform. The device ablates whilst continuously irrigating with saline and aspirating dissolved tissue, which we found improved the surgeon's view of operated structures. There was minimal blood contamination, and coblation reduced the need for repeated instrumentation in achieving final haemostasis.

From a patient perspective, this minimally invasive procedure was associated with favourable outcomes. The direct transnasal access produced no visible scars and facilitated local dissection that avoided overtreatment, with no unnecessary resection of the normal tissue. The hospital stay required was minimal, and the patient could promptly return to his normal daily activities.

No tumour recurrences were reported amongst the previously published cases, after a mean follow-up interval of 24.7 months (range 6–52 months) [3–11]. We do not anticipate tumour recurrence in our patient's case; however, should unexpected recurrence be encountered, then no future surgical treatment options would be compromised because of his primary resection procedure.

We have documented the coblator-assisted resection of a large nasopharyngeal pleomorphic adenoma. The endoscopic transnasal technique used was straightforward to perform, and we found that use of a coblator offered the surgeon clear visualisation of the operative field during dissection whilst facilitating excellent haemostasis. The procedure was well tolerated and had favourable patient outcomes. This is a useful addition to the existing literature on the surgical procedures used to treat benign pathology in the postnasal space. Although further studies will be necessary to establish technique reproducibility and limitations, with careful case selection, we suggest that a coblator-assisted resection could be considered when approaching similar lesions in future.

## Figures and Tables

**Table 1:** Summary of published reports of nasopharyngeal pleomorphic adenoma.

Reference (year)	Age (years)	Sex (M/F)	Presenting symptoms	Tumour size (mm)	Preoperative biopsy	Diagnostic imaging	Surgical approach	Surgical complications	Outcome
Yumoto et al. [2] (1992)	54	F	—	—	—	—	Transmandibular transpterygoid	Soft palate paresis and nasal speech	—

Roh et al. [3] (2005)	61	F	Nasal obstruction; epistaxis	30 × 30 × 20 (macroscopic)	PA	CT/MR	Endoscopic transnasal/transoral	None	No recurrence at 2 years

Lee et al. [4] (2006)	78	M	Hearing loss; aural fullness; otalgia; pulsatile tinnitus	—	—	—	Endoscopic-guided	Eustachian tube dysfunction	No recurrence at 20 months

de Almeida et al. [5] (2009)	66	F	Nasal obstruction; voice change; dysphagia	55 × 40 (macroscopic)	PA (FNA)	MR	Endoscopic transnasal/transoral	None	No recurrence at 2 years

Thakur et al. [6] (2010)	35	M	Nasal obstruction; hearing loss; aural fullness; voice change; dysphagia	—	? SCC (FNA) *later biopsy showed* PA	CT	Transpalatal	None	No recurrence at 1 year

Martínez- Capoccioni et al. [7] (2012)	52	F	Nasal obstruction; hearing loss; aural fullness; otalgia; tinnitus	30 × 25 (macroscopic)	—	CT	Endoscopic transnasal/transoral	None	No recurrence at 52 months

Pagella et al. [8] (2012)	57	F	Nasal obstruction	28 × 18 × 18 (macroscopic)	—	CT	Endoscopic transnasal/transoral	None	No recurrence at 4 years

Berrettini et al. [9] (2013)	51	F	Nasal obstruction; hearing loss; aural fullness; otalgia	30 × 40 × 30 (endoscopic)	PA	MR	Endoscopic transnasal	—	No recurrence at 6 months

Maruyama et al. [10] (2014)	80	F	Hearing loss	21 × 19 × 19 (macroscopic)	PA	CT/MR	Endoscopic transnasal/transoral	None	No recurrence at 2 years

Yazici et al. [11] (2015)	62	M	Hearing loss; aural fullness; otalgia	13 × 20 × 13 (imaging)	PA	MR	Endoscopic transnasal	None	No recurrence at 1 year

Grech et al. [12] (2017)	29	F	Nasal obstruction; aural fullness; voice change; snoring	39 × 51 × 33 (imaging)	PA	CT/MR	Transoral	None	—

Present case	39	M	Nasal obstruction; otalgia; voice change	35 × 32 × 25 (macroscopic)	PA	CT/MR	Endoscopic transnasal/transoral	None	No recurrence at 1 year

M, male; F, female; PA, pleomorphic adenoma; FNA, fine needle aspiration; SCC, squamous cell carcinoma; CT, computed tomography; MR, magnetic resonance.
